# In Vivo Assessment of Hepatic and Kidney Toxicity Induced by Silicon Quantum Dots in Mice

**DOI:** 10.3390/nano14050457

**Published:** 2024-03-01

**Authors:** Roxana-Elena Cristian, Cornel Balta, Hildegard Herman, Bogdan Trica, Beatrice G. Sbarcea, Anca Hermenean, Anca Dinischiotu, Miruna S. Stan

**Affiliations:** 1 Departament of Biochemistry and Molecular Biology, Faculty of Biology, University of Bucharest, 91-95 Splaiul Independentei, 050095 Bucharest, Romania; roxana.cristian@drd.unibuc.ro (R.-E.C.); anca.hermenean@gmail.com (A.H.); miruna.stan@bio.unibuc.ro (M.S.S.); 2 DANUBIUS Department, National Institute of Research and Development for Biological Sciences, Splaiul Independentei 296, 060031 Bucharest, Romania; 3 “Aurel Ardelean” Institute of Life Sciences, Vasile Goldis Western University of Arad, 86 Rebreanu, 310414 Arad, Romania; balta.cornel@uvvg.ro (C.B.); herman.hildegard@uvvg.ro (H.H.); 4 National Institute for Research & Development in Chemistry and Petrochemistry (INCDCP-ICECHIM), 202 Spl. Independentei, 060021 Bucharest, Romania; trica.bogdan@gmail.com; 5 Materials Characterization Department, National Institute for Research & Development in Electrical Engineering (ICPE-CA), 313 Splaiul Unirii, 030138 Bucharest, Romania; gabriela.sbarcea@icpe-ca.ro; 6 Research Institute of the University of Bucharest (ICUB), University of Bucharest, 91-95 Spl. Independentei, 050095 Bucharest, Romania

**Keywords:** silicon quantum dots, mice, hepatic and renal toxicity, oxidative stress, histones

## Abstract

In the last decade, silicon-based quantum dots (SiQDs) have attracted the attention of researchers due to their unique properties for which they are used in medical applications and in vivo imaging. Detection of cytotoxic effects in vivo is essential for understanding the mechanisms of toxicity, a mandatory step before their administration to human subjects. In this context, we aimed to evaluate the in vivo hepatic and renal acute toxicity of SiQDs obtained by laser ablation. The nanoparticles were administrated at different doses (0, 1, 10, and 100 mg of QDs/kg of body weight) by intravenous injection into the caudal vein of Swiss mice. After 1, 6, 24, and 72 h, the animals were euthanatized, and liver and kidney tissues were used in further toxicity tests. The time- and dose-dependent effects of SiQDs on the antioxidant defense system of mice liver and kidney were investigated by quantifying the activity of antioxidant enzymes (catalase, superoxide dismutase, glutathione peroxidase, glutathione reductase, and glutathione S-transferase) in correlation with the morphological changes and inflammatory status in the liver and kidneys. The results showed a decrease in the activities of antioxidant enzymes and histopathological changes, except for superoxide dismutase, in which no significant changes were registered compared with the control. Furthermore, the immunohistochemical expression of TNF-α was significant at doses over 10 mg of QDs/kg of body weight and were still evident at 72 h after administration. Our results showed that doses under 10 mg of SiQDs/kg of b.w. did not induce hepatic and renal toxicity, providing useful information for further clinical trials.

## 1. Introduction

Quantum dots (QDs) are crystalline, semiconducting particles with unique optical and spectroscopic properties, such as superior resistance to photooxidation and strong fluorescence emission, which are characteristics that are dependent on particle size, shape, and composition. In addition, the simultaneous excitation of several fluorescence levels leads to different colors depending on the size of the nanoparticles [1,2,3]. 

Silicon-based QDs (SiQDs) are a class of NPs with special properties, such as low toxicity and easy-to-modify surface properties [4]. For this reason, they are used in applications such as bioimaging, biosensors [5,6], fluorescent labeling, drug administration, protein detection techniques, tissue engineering [7,8], and photodynamic therapies (used in the treatment of skin diseases) [9]. 

The synthesis of QDs can be achieved either by chemical methods offering the advantage of large-scale production of nanoparticles and control of material purity or by physical methods obtaining high-purity particles of the desired size. However, both types of methods also have disadvantages. QDs obtained by physical methods have a lower yield, whereas in the case of chemical methods, the purity of the material is challenging to achieve. There are several methods of QD synthesis: laser ablation, electrochemical etching, microwave-assisted method, etc. The laser ablation method has the advantage of it not requiring chemical precursors and ensuring sterility, and the experimental conditions are easy to obtain without the need for high temperatures or very high pressures. In addition, the method is versatile, as experimental conditions may vary [2].

Despite the many properties and wide range of applicability, many researchers are concerned about the possible toxicity of QDs [10]. The main challenge faced in using QDs is the lack of information about their safety and how they affect the biological integrity of organisms by producing toxicological, cytotoxic, and genotoxic effects [11]. SiQDs’ potential to induce an inflammatory response was evaluated on the MRC-5 human lung fibroblasts, where it was revealed that QDs affected the membrane integrity, as indicated by the time- and dose-dependent increase in lactate dehydrogenase levels in the cell culture media, as well as the accumulation of autophagosomes and increase in the lysosome levels. Moreover, cell morphology was affected, as microscopy images showed broken actin filaments. Inflammatory processes were detected at the cellular level, as demonstrated by the increase in nitric oxide and interleukin-6 levels and the appearance of redox imbalances in the turnover of extracellular matrix through a different regulation of matrix metalloproteinases and their inhibitors [12]. Analysis of the redox state at the cellular level indicated an increase in the level of reactive oxygen species (ROS), a decrease in reduced glutathione (GSH) concentration, and the formation of a strongly oxidized environment through the accumulation of oxidized proteins [13]. Moreover, recent studies [14] proved that these SiQDs possess significant immunotoxicity on murine RAW 264.7 macrophages.

Biodistribution and in vivo effects of SiQDs were analyzed after intraperitoneal injection in carp. The presence of QDs in the liver was observed by fluorescence microscopy for at least two weeks after exposure. Moreover, the assessed markers of oxidative stress indicated the onset of oxidative stress in liver and the onset of antioxidant defense mechanisms such as increased levels of antioxidant enzymes, recovery of GSH concentration, and activation of the chaperone proteins Hsp30 and Hsp70. In the same study, the ability of SiQDs to induce an inflammatory response was demonstrated, as confirmed by the profiles of heat shock proteins (Hsp60 and Hsp90). However, by activating cyclooxygenase COX-2, the profile of matrix metalloproteinases indicated a remodeling process in the liver three weeks after administration [15]. 

Another study evaluated the effects of microinjection of water-soluble SiQDs in the animal pole region of *Danio rerio* (zebrafish) embryos using fluorescence microscopy, and it revealed that the survival rate of embryos depended on the dose of nanoparticles used, the most common changes induced being edema and tail truncation [16]. Investigation of the near-infrared QDs administered intravenously to BALB/C mice at the dose required for in vivo tumor imaging reported a lack of liver and kidney toxicity [17]. Also, an evaluation of the hepatotoxicity of Mn-doped ZnS QDs and polyethylene glycol-coated derivatives (up to 5 mg/kg of body weight (b.w.)) administered by injection in male Kunming mice indicated no obvious liver damage [18]. Moreover, the evaluation of CdTe QDs effects on the antioxidant activity of liver and kidney indicated the ability of these nanoparticles to reduce the GSH level and the capacity to scavenge hydroxyl and superoxide radicals, inducing oxidative tissue damage [19]. Exposure to CdTe QDs by intravenous administration to mice showed increased hepatic lipid peroxidation levels [20].

Studying how nanoparticles interact with biological systems is a critical step in developing the range of applicability of these materials. Furthermore, understanding the mechanisms triggered by nanoparticles at the biological level generates the ability to develop nanomaterials with new surface properties and a high degree of biocompatibility [21]. Therefore, our study aimed to obtain an in vivo toxicological evaluation of SiQDs by evaluating the degree of oxidative stress associated with morphological changes and inflammatory status in the liver and kidneys. 

## 2. Materials and Methods

### 2.1. Synthesis of SiQDs and Their Characteristics

The SiQDs used within this study were obtained at the National Institute for Laser, Plasma and Radiation Physics, Bucharest, Magurele, Romania, by the method described in 2005 by Grigoriu et al. using ablation in a stainless steel chamber [22]. Briefly, the laser beam was focused on a silicon target inside the chamber; the nanoparticles were taken up by the gas flow and directed to a Millipore filter with a pore size of 100 nm, reacted with oxygen in the air, formed a silicon dioxide layer on their surface, and resulted in core/shell QDs of silicon/silica.

The SiQDs were analyzed by scanning electron microscopy (SEM) on a Zeiss Auriga microscope (Carl Zeiss, Jena, Germany), acquiring the images at a voltage of 5 kV, and in a bright-field mode of transmission electron microscopy (TEM) using a Tecnai F20 G2 TWIN Cryo-TEM (FEI, Hillsboro, OR, USA) at an acceleration voltage of 200 kV.

The measurement of the hydrodynamic size, polydispersity index, and zeta potential of SiQDs dispersed in saline was performed in triplicate at 25 °C using a refractive index of 1.52 on a Malvern Nano-ZS instrument (Malvern Instruments, Malvern, Worcestershire, UK). The absorbance and emission spectra of SiQDs were recorded in 0.9% NaCl solution using the FlexStation 3 reader (Molecular Devices, San Jose, CA, USA).

### 2.2. Administration of SiQDs to Mice

Adult male Swiss mice (~25 g/animal) were housed in the Animal facility of Western University “Vasile Goldiș” of Arad in compliance with EU and national regulations (Ethical Committee Approval no. 14 from 27 October 2015). The mice received a standard diet (pellets and water ad libitum), temperature (20–25 °C), and circadian rhythm (12 h light/12 h darkness). These conditions were maintained throughout the experiment. Subsequently, the mice were randomly distributed into four groups, corresponding to each time interval (1 h, 6 h, 24 h, and 72 h). Each group contained 4 subgroups of 5 mice: control that received saline, 1 mg of QDs/kg of b.w., 10 mg of QDs/kg of b.w. and 100 mg of QDs/kg of b.w. The treatments were injected using a syringe into the caudal vein. The mice corresponding to each group were euthanatized at 1 h, 6 h, 24 h, and 72 h post-injection of QDs, and the organs selected for this study (liver and kidneys) were preserved according to biochemistry and histology protocols. No significant differences regarding the weight of liver and kidneys between the control and the other experimental groups of mice were noticed.

### 2.3. Histology and Immunohistochemistry

Liver and kidney samples were fixed in a 4% formaldehyde solution in PBS, embedded in paraffin, and stained using routine hematoxylin and eosin stain (H&E). An Olympus BX43 microscope with a digital camera (Olympus XC30, Olympus, Hamburg, Germany) was used for the microscopy analyses of the slides. Prior to immunochemistry, the sections were deparaffinized and rehydrated. The primary antibody used was TNF-α (1:200 dilution), and then specific secondary antibodies were applied. The immunoreaction was detected using a Novocastra Peroxidase/DAB kit (Leica Biosystems, Nussloch, Germany). The negative control sections were stained with irrelevant immunoglobulins and analyzed under a bright-field microscope. After dehydration, tissue sections were mounted with BioMount mounting medium and analyzed by light microscopy using the Olympus System Microscope Model BX43 equipped with a digital camera.

### 2.4. Obtaining the Tissue Homogenates

The tissue homogenates for each biological sample were obtained by weighing 0.1 mg of tissue and adding 1 mL of 0.1 M TRIS-HCl–5 mM EDTA buffer (pH = 7.4). The homogenization was performed three times for 2 min using a mixer mill (Retsch MM301, Retsch GmbH, Haan, Germany) at a frequency of 30 vibrations/s. The tissue suspension was centrifuged at 10,000 rpm for 10 min at 4 °C, and the supernatant was collected and kept at −80 °C. The total protein content was measured using the Folin reagent-based method.

### 2.5. Measurement of Antioxidant Enzyme Activities

Using a method outlined by Aebi [23], the decrease in H_2_O_2_ concentration recorded at 240 nm was used for the measurement of catalase (CAT) activity. Superoxide dismutase (SOD) activity was measured based on NADH oxidation, which was demonstrated by a decrease in the absorbance at 340 nm [24]. Using GSH and tert-butyl hydroperoxide as substrates, the activity of glutathione peroxidase (GPx) was measured at 340 nm [25]. For the reaction catalyzed by glutathione reductase (Gred), the changes in absorbance intensity at 340 nm were recorded after the conversion of NADPH to NADP+ as a result of the reduction of GSSG to GSH [26]. Furthermore, the rate of 1-chloro-2,4-dinitrobenzene (CDNB) conjugation with GSH was used to measure the activity of glutathione S-transferase (GST) [27]. The intensity of absorbance was read on a JASCO V-530 spectrophotometer. The enzyme activities were expressed in terms of units of activity per milligram of protein (U/mg) and as a percentage from the control.

### 2.6. Measurement of Malondialdehyde Level (MDA)

The MDA level was determined based on a standard solution of 1,1,3,3-tetramethoxypropane. A volume of 700 µL of 0.1 M HCL was pipetted over the MDA calibration curve and the cell lysates. The mixtures were homogenized by vortexing and left to stand at room temperature for 20 min. After that, a volume of 900 μL of thiobarbituric acid (0.025 M concentration) was added to all tubes and incubated for 65 min at 37 °C in order to form the TBA-MDA products. Subsequently, the fluorescence intensity recorded on a Jasco FP-6300 fluorimeter was converted to nmol MDA using the standard curve.

### 2.7. Measurement of GSH Level 

The GSH level was determined with the Glutathione Assay Kit from Sigma-Aldrich (Burlington, MA, USA) according to the protocol provided by the manufacturer. The method involved the reduction of 5,5′-dithiobis-2 nitrobenzoic acid (DTNB) by GSH with the formation of 5-thio-2 nitrobenzoic acid (TNB). Cell lysates were diluted accordingly and deproteinized with 5% 5-sulfosalicylic acid. This was followed by centrifugation at 3000× *g* for 5 min at 4 °C to remove precipitated proteins. A volume of 10 µL supernatant was mixed with 150 µL of assay buffer solution with DTNB (1.5 mg/mL). The absorbance of the yellow product obtained from this reaction was measured at 412 nm on a Tecan Genios spectrophotometer (Tecan, Männedorf, Switzerland). An extrapolation on a curve with a GSH standard was performed, and the results were expressed in nmol/mg of protein.

### 2.8. Western Blot

The protein level of p53, Beclin-1, LC-3, and Nrf-2 was determined in the samples collected from the control and 100 mg of SiQD/kg of b.w. after 1, 6, 24, and 72 h. Cell lysates corresponding to 100 µg of protein were separated on a 10% SDS/PAGE under reducing conditions and transferred to 0.4 µm polyvinylidene difluoride (PVDF) membrane in a wet transfer tank (Bio-Rad, Hercules, CA, USA). Membranes were blocked with the blocking solution included in the WesternBreeze Chromogenic kit (Invitrogen, Rockford, IL, USA) for 30 min at room temperature. The detection of p53, LC-3, and Nrf-2 proteins was performed with the rabbit polyclonal anti-p53, anti-LC-3, and anti-Nrf-2 primary antibodies, respectively (1:250 dilution). The highlight of Beclin-1 protein was performed using anti-Beclin-1 mouse polyclonal primary antibody (1:250 dilution, SantaCruz Biotechnology, Dallas, TX, USA). After the incubation with primary antibodies, membranes were processed according to the manufacturer’s instructions, using alkaline phosphatase-conjugated anti-mouse and anti-rabbit secondary antibodies and 5-bromo-4-chloro-3′-indolephosphate/nitroblue tetrazolium as the chromogenic substrate. The resulting bands were visualized and photographed using a transilluminator (ChemiDoc MP Video Documentation System, Bio-Rad, Hercules, CA, USA) and were analyzed using the GelQuant.NET software version 1.7.8.

### 2.9. Detection of 8-Hyroxy-2′-deoxyguanosine (8-OHdG)

8-OHdG is the best known and most widely used biomarker to evaluate the damage produced at the level of nucleic acids following the oxidative stress induced by nanoparticles [28]. 

DNA extraction was performed with the PureLink^®^ Genomic DNA Kit (Invitrogen, Waltham, MA, USA). The measurement of the relative level of 8-OHdG was performed using an ELISA kit (Abcam, Cambridge, UK) in murine liver and kidney tissues collected after 72 h from the administration of SiQDs (100 mg/kg of b.w.). Murine tissue samples were pooled for all 5 individuals of the same group. This technique was previously indicated for molecular biology tests, presenting the advantage of reducing inter-individual variations and providing an overview of the analyzed group [29,30]. 

### 2.10. Quantification of 5-methyl cytosine (5-mC)

DNA methylation plays an essential role in maintaining genome stability, but methylation patterns are susceptible to changes in response to environmental factors. Aberrant methylation has been detected in several diseases, including cancer [31]. Global DNA methylation can be assessed by measuring the level of 5-mC using the ELISA technique in murine liver and kidney tissue samples collected after 72 h of post-administration of SiQDs (100 mg/b.w.) with the 5-mC DNA ELISA Kit (Enzo Sciences, Farmingdale, NY, USA). This method was carried out based on the same extracts previously mentioned for the detection of 8-OHdG, with the mention that the volumes’ standardization was carried out according to the kit’s instructions.

### 2.11. Assessment of Histone Methylation

H3 and H4 histones have long tails that protrude from the nucleosome and can be covalently modified (by methylation, acetylation, phosphorylation, etc.) at multiple sites. These changes impact gene regulation, DNA repair processes, and chromatin condensation in mitosis. Firstly, the extraction of total histones was performed using the EpiQuik™ Total Histone Extraction Kit (Epigentek, Farmingdale, NY, USA), followed by the determination of protein concentration using the Bradford reagent. Next, the detection of histone H4 modification was performed using the EpiQuik™ Histone H4 Modification Multiplex Assay Kit (Epigentek, Farmingdale, NY, USA).

### 2.12. Statistical Analysis

All tests were statistically analyzed using Student’s *t*-test (Microsoft Excel version 10) and are expressed as the mean value ± standard deviation (SD) (*n* = 3). A *p* value of less than 0.05 was considered statistically significant.

## 3. Results

### 3.1. Physicochemical Characteristics of SiQDs

**Figure 1:**
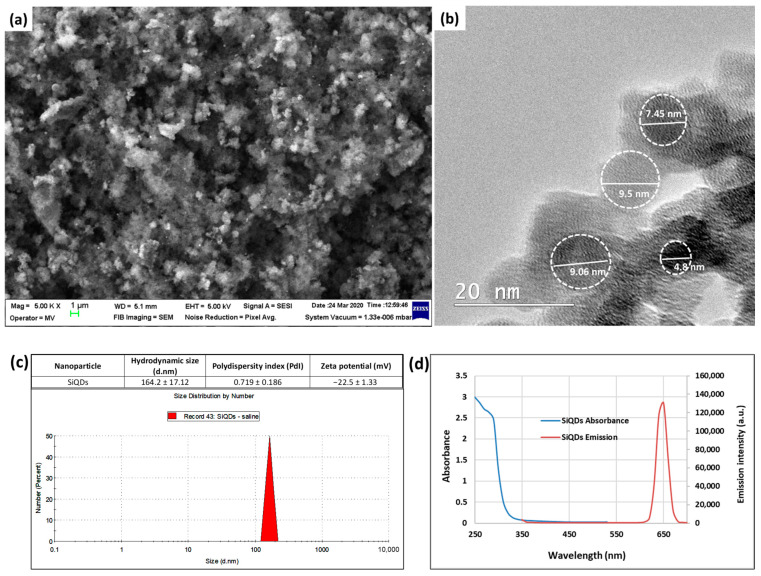
Morphological characterization of SiQDs by SEM (**a**) and TEM (**b**) investigations, with the spherical shape of QDs being marked by white dot circles. Hydrodynamic size, polydispersity index, and zeta potential (**c**) and absorbance and emission spectra (**d**) were measured for SiQDs dispersed in 0.9% NaCl solution.

The SEM image (Figure 1a) revealed clusters of nanoparticles, confirming the aggregation tendency of SiQDs, although the average size for each individual particle was under 10 nm in the TEM image (Figure 1b). The dispersion in saline did not change the aggregation pattern of SiQDs, which was previously observed in water [32], with the hydrodynamic size being around 165 nm and the polydispersity index being higher than 0.72 (Figure 1c). Furthermore, the fluorescence of SiQDs was proven by the emission spectrum, with a peak at 650 nm after the excitation at 325 nm (Figure 1d).

### 3.2. Histopathology and Immunohistochemical Analysis of the TNF-α in the Mice Liver and Kidneys Exposed to SiQDs

**Figure 2:**
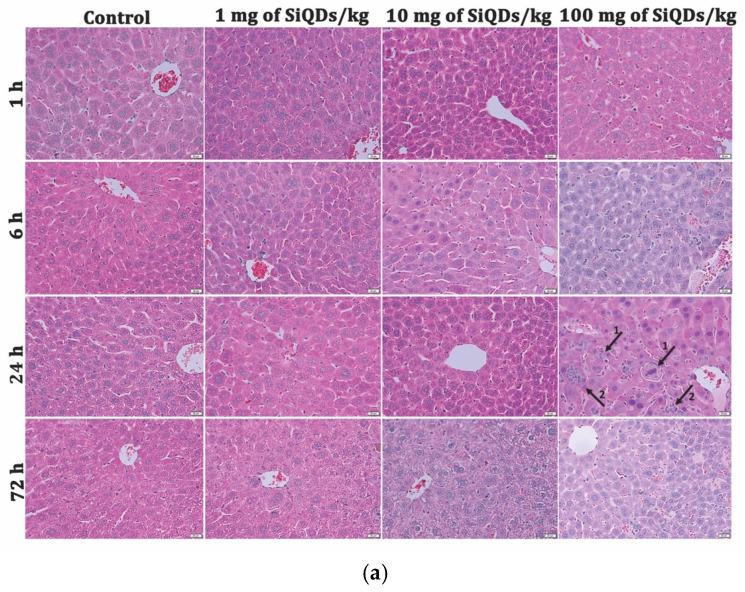

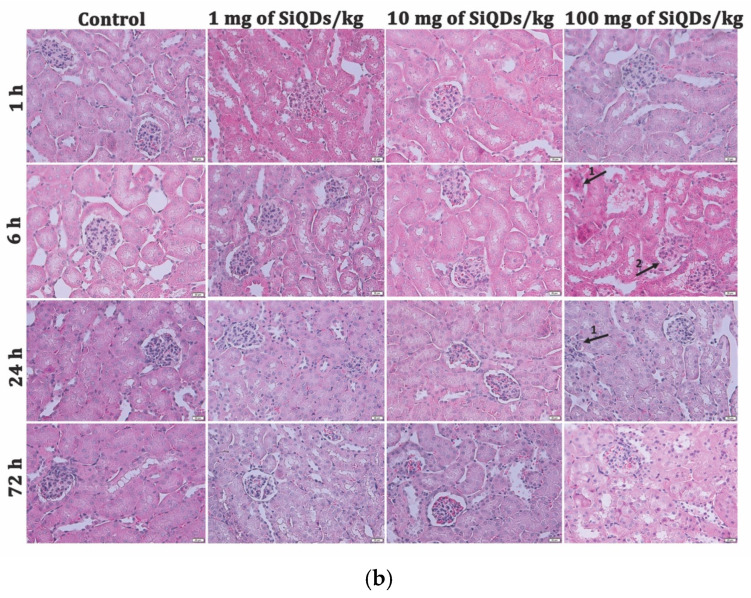
Liver (**a**) and kidney (**b**) histopathology at 1 h, 6 h, 24 h, and 72 h after SiQDs administration (1, 10, and 100 mg/kg of b.w.) in mice. Tissue sections (5 µm) were stained with hematoxylin and eosin and examined by light microscopy. Legend of arrows: (**a**) 1—hepatocyte swelling, 2—inflammatory infiltrates; (**b**) 1—vascular congestion; 2—glomerular atrophy. Magnification of 10× for liver images and 20× for kidney images.

The control of liver and kidney tissues exhibited typical histological structures at all intervals (Figure 2). The hepatocytes formed well-arranged cords, normal hepatic sinusoids, and parenchyma without inflammatory cell infiltration or necrosis. The glomerular and renal tubular structures have typical structures without abnormal pathological findings. After exposure to the SiQDs, the mice of experimental groups exhibited significant changes in the liver and kidney tissue morphology, and the alterations gradually increased in a dose-dependent manner, starting at over 6 h after injections, and slightly decreased at 72 h. Significant liver damage was noticed in the mice that received the high dose of 100 mg SiQDs/kg of b.w. This liver pathology was characterized by structural changes that included hydropic degeneration, swelling of hepatocytes, and the presence of inflammatory cells, predominantly located around the central veins and portal spaces (Figure 2a). Similarly, the kidney exhibited vascular congestion and glomerular atrophy in a dose-dependent manner (Figure 2b). 

**Figure 3:**
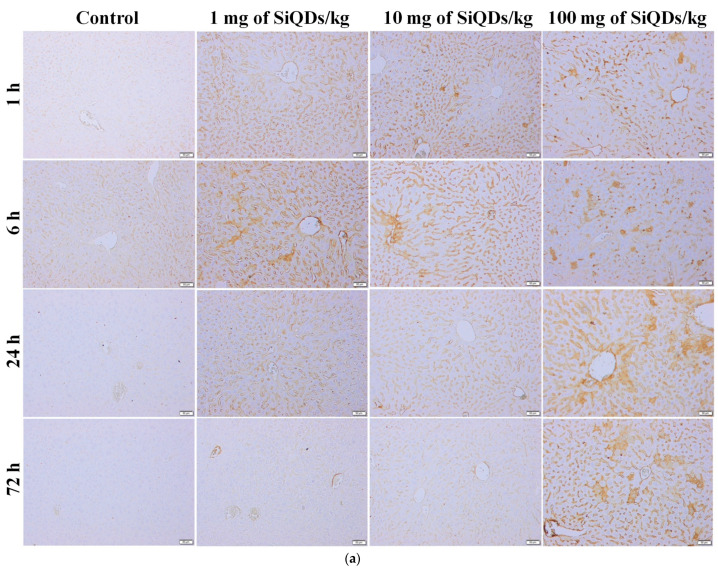

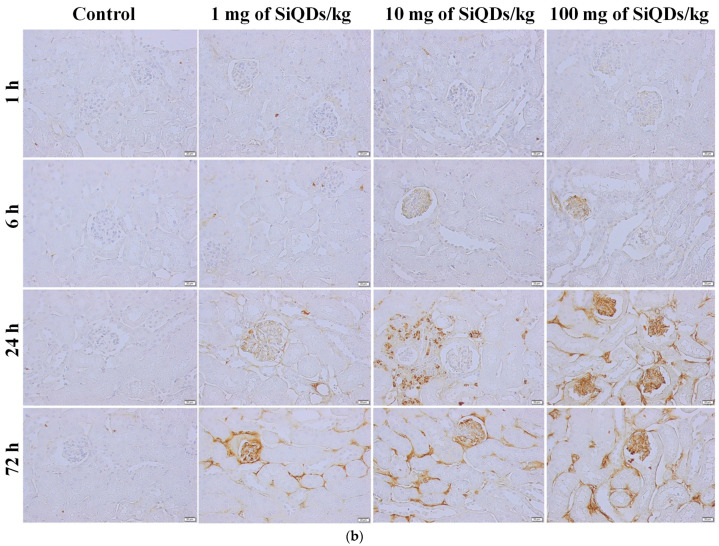
Immunohistochemistry for TNF-α in the liver (**a**) and kidneys (**b**) at 1 h, 6 h, 24 h, and 72 h after SiQDs administration (1, 10, and 100 mg/kg of b.w.) in mice. Magnification of 10× for liver images and 20× for kidney images.

Immunohistochemical analyses showed immunopositivity for TNF-α starting at 6 h after the SiQDs administration for both organs, mainly for the highest dose of 100 mg/kg of b.w. (Figure 3). 

### 3.3. Analysis of Oxidative Stress Induced by SiQDs Administration

**Figure 4:**
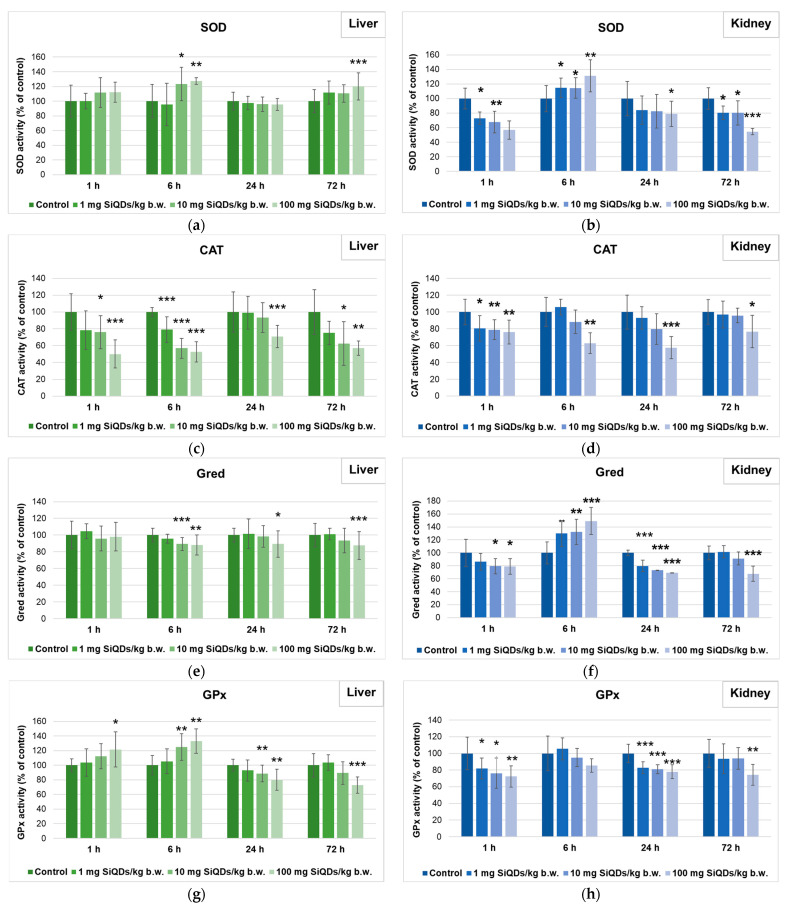

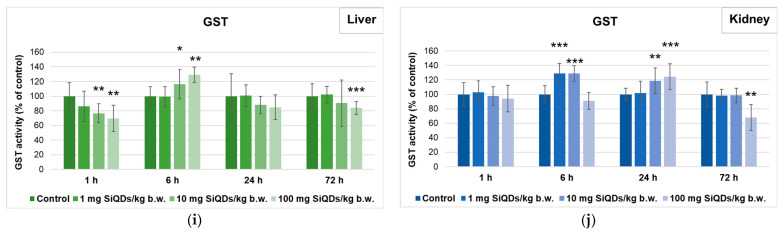
Specific activities of SOD (**a**,**b**), CAT (**c**,**d**), Gred (**e**,**f**), GPx (**g**,**h**), and GST (**i**,**j**) in liver (**a**,**c**,**e**,**g**,**i**) and kidney (**b**,**d**,**f**,**h**,**j**) tissues collected at 1, 6, 24, and 72 h after SiQDs administration. Results are calculated as the mean ± SD (*n* = 5) and are represented relative to the control. * *p* < 0.05, ** *p* < 0.01, and *** *p* < 0.001 compare with the control.

The results obtained after measuring the specific activity of SOD in liver samples (Figure 4a) indicated that no excessive amount of superoxide anion was generated inside the mouse hepatocytes after SiQDs administration. Although an initial increase with 27% above the control was noticed in the case of the highest concentration of QDs after 6 h, later, the SOD activity values returned to levels almost similar to those of the control, indicating the neutralization of the oxidative effects produced by the SiQDs. In the case of kidney tissue (Figure 4b), a dose-dependent decrease in this enzyme activity was observed after the first hour. Further, it was recorded that there was an increase of 31% above the control in the 6 h group and a decrease by 45% compared with the control in the 72 h group that received 100 mg of SiQDs/kg of b.w., which indicated a higher sensitivity of the kidney to SiQDs. 

As shown in Figure 4c, the level of CAT activity in the liver of mice that received SiQDs underwent a significant dose-dependent reduction, with the catalase activity level reaching about half of the control value after all exposure periods. In the kidney samples (Figure 4d), a significant dose-dependent decrease in CAT activity was also observed, the lowest level being recorded in the case of 100 mg of SiQDs/kg of b.w. at 24 h (by 43% lower compared with the control level).

As shown in Figure 4e, the level of Gred activity recorded a slight decrease, the lowest value being 13% lower than the control in the 72 h group exposed to 100 mg of SiQDs/kg of b.w. In the case of kidneys (Figure 4f), a dose-dependent increase in the enzyme activity for the 6 h group and a decrease after 24 and 72 h was noticed, the lowest value recorded being 32% lower than the control in the 72 h group that received 100 mg of SiQDs/kg of b.w. 

In the case of liver tissue (Figure 4g), GPx activity increased depending on concentration in the 1 h and 6 h groups, with an increase of 133% compared with the control after 6 h. These data indicated the stimulation of antioxidant activity at the cellular level, demonstrating that the oxidative stress induced by SiQDs increased the antioxidant capacity of cells. In the case of the 24 and 72 h groups, a slight decrease in GPx activity compared with the control was registered for the highest QDs dose. Furthermore, a reduction in a dose-dependent manner of the GPx activity was observed in the kidney tissue after all time periods (Figure 4h).

Regarding the GST activity, a dose-dependent decrease was observed in the liver after 1 h (Figure 4i), in agreement with the same pattern of CAT activity reduction. Further, the dose-dependent increase in GST activity in liver after 6 h correlated very well with the same trend of GPx activity. For the next time intervals tested, 24 and 72 h, GST activity did not change too much compared with the control, resembling that of the activities of SOD, Gred, and GPx, except for the maximal dose at 72 h. In the case of the kidneys, the dynamics of GST activity over time showed the highest decrease compared with the control after 72 h for the group of mice that received 100 mg of SiQDs/kg of b.w. (Figure 4j). 

**Figure 5:**
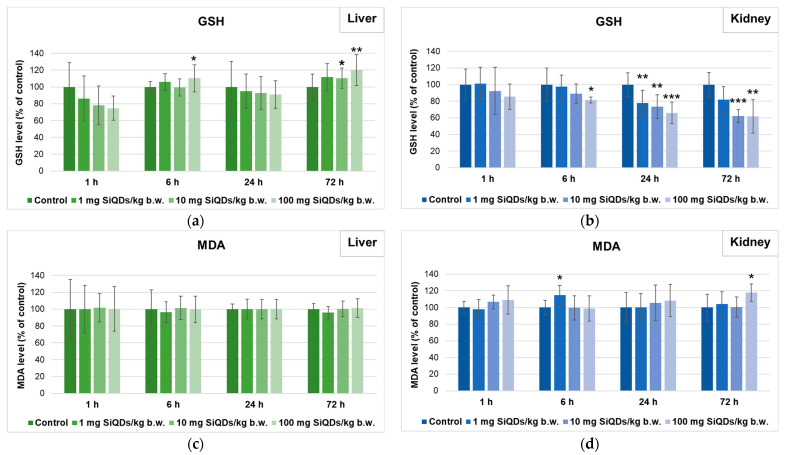
Levels of GSH (**a**,**b**) and MDA (**c**,**d**) in liver (**a**,**c**) and kidney (**b**,**d**) tissues collected at 1, 6, 24, and 72 h after SiQDs administration. Results are calculated as the mean ± SD (*n* = 5) and are represented relative to the control. * *p* < 0.05, ** *p* < 0.01, and *** *p* < 0.001 compare with the control.

Regarding the most important non-enzymatic antioxidant, GSH, a decrease by 25% compared with the control was initially assessed after 1 h in the liver of mice that received 100 mg of SiQDs/kg of b.w. Further, the level of GSH increased, suggesting the recovery of this antioxidant at the cellular level (Figure 5a). In contrast, in the kidney tissue, a dose- and time-dependent decrease in the GSH level was observed (Figure 5b). The minimum level of GSH was recorded in the case of the 72 h group of 100 mg of SiQDs/kg of b.w. (a significant decrease of 38% compared with the control).

The effect of SiQDs on lipid peroxidation was evaluated throughout the MDA level. In the liver (Figure 5c), only an increase in the MDA level of 35% above the control for the 6 h group of 10 mg of SiQDs/kg of b.w. was registered, with the MDA values being close to those of the control for the other groups. Also, a slight increase in MDA levels was noticed in the kidney tissue of the 1 h, 24 h, and 72 h groups injected with the highest dose of QDs and after 6 h in the case of 1 mg/kg of b.w. (Figure 5d).

### 3.4. Analysis of Proteins’ Expression Involved in the Antioxidant Defense System, Apoptosis, and Autophagy

**Figure 6:**
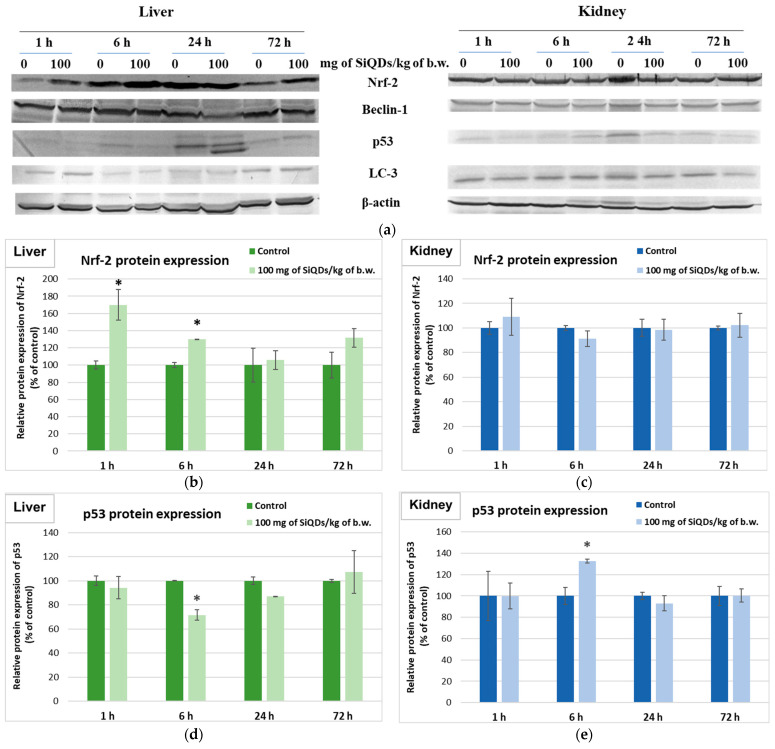

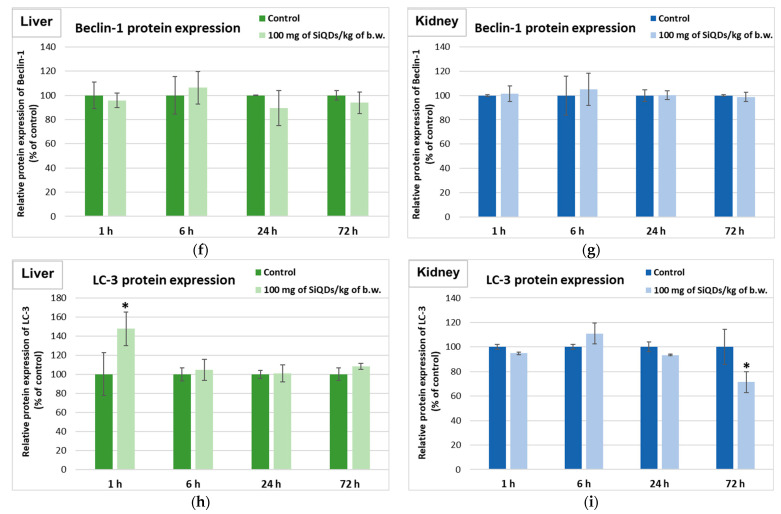
Changes in the expression of proteins involved in the antioxidative defense response, apoptosis, and autophagy after SiQDs administration (100 mg/kg of b.w.) in mice. The analysis of Nrf-2, p53, Beclin-1, and LC-3 protein expression by Western blotting (**a**) was quantified in the liver (**b**,**d**,**f**,**g**) and kidney (**c**,**e**,**g**,**i**) tissues collected at 1, 6, 24, and 72 h after SiQDs administration. Results are calculated as the mean ± SD (*n* = 5) and are represented relative to the control. * *p* < 0.05 compares with the control.

The influence of SiQDs on various cell signaling pathways involved in the response of hepatic and renal cells to oxidative stress was assessed throughout the measurement of Nrf-2, p53, Beclin-2, and LC-3 proteins’ expression (Figure 6a). The Nrf-2 signaling pathway controls the expression of genes that encode proteins involved in detoxification, and it also controls the elimination of reactive species through conjugation reactions [33]. The Nrf-2 protein level increased by 70% above the control in the liver samples of the 1 h group (Figure 6b). Although the expression of Nrf-2 was upregulated up to 72 h in hepatic cells, there were no changes compared with the control in the kidneys (Figure 6c). 

The p53 protein is the most well-known factor relevant to the prevention of malignancy, having as its main functions the induction of apoptosis and arrest of the cell cycle. Also, p53 is involved in DNA repair, the control of metabolic pathways, and driving cells into senescence [34]. After one hour of exposure to 100 mg of SiQDs/kg of b.w., the expression of p53 in liver and kidneys of mice was unchanged compared with the control. A decrease of 28% in its relative expression compared with the control was noted in liver, and an increase by 33% compared with the control was measured in kidney tissues at the first 6 h after administration. However, p53 expression returned to values close to the control after 24 and 72 h (Figure 6d,e).

The SiQDs’ ability to induce autophagy in mouse liver and kidney tissues after exposure to 100 mg of SiQDs/kg of b.w. was evaluated by quantifying the Beclin-1 and LC-3 proteins’ expression (Figure 6e–h). Beclin-1 is a component of the nuclear complex called the phagophore that is involved in the early stages of autophagosome synthesis. No important changes in comparison with the control all over the 72 h experiment were noticed, regardless of the tissue tested. LC-3 is conjugated with phosphatidyl amine following the induction of autophagy and is involved in the formation of the autophagosomes’ membrane. Regarding the dynamics of LC-3 protein expression over time, an increase by 48% above the control was noticed after 1 h in liver, but after 6, 24, and 72 h, its level remained at the control level. Also, in kidneys, the exposure to 100 mg of SiQDs/kg of b.w. did not change the LC-3 protein expression after 1, 6, and 24 h compared with the control, but a decrease of 29% from the control in the case of kidney samples after 72 h was registered.

### 3.5. Genotoxicity Evaluation

#### 3.5.1. Analysis of the 8-OHdG Level

**Figure 7:**
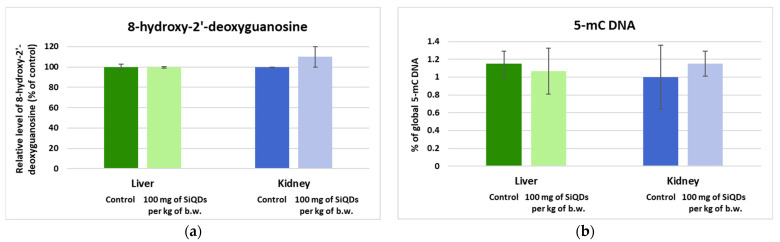
The levels of 8-OHdG (**a**) and global DNA methylation (**b**) as determined by the ELISA technique in the murine liver and kidney samples collected at 72 h after the administration of SiQDs (100 mg of QDs/kg of b.w.). Results are expressed as the mean ± SD (*n* = 5).

The level of 8-OHdG is of high biological importance because this compound can induce guanine to thymine transversions, representing the most frequent somatic mutations discovered in human cancer [35]. Increases in the level of 8-OHdG have been reported after exposure to nanoparticles, which can cause DNA damage and cell death [36]. According to our data, the percentage of 8-OHdG in the mice liver exposed to 100 mg of SiQDs/kg of b.w. remained at the same level as in the control group, but in the case of the kidneys, a slight increase (110% of control) was recorded (*p* < 0.05), which indicated that the kidney is more sensitive to the toxic effects of SiQDs compared with the liver (Figure 7a).

The most frequent site where the covalent binding of the methyl group takes place in the DNA of mammals is represented by the carbon 5 atom of cytosine. This chemical reaction can be triggered by exposure to heavy metals such as cadmium, nanoparticles, or radiation [37]. Global DNA methylation can be estimated throughout the level of 5-mC [38]. Our results showed no significant changes compared with the control for both types of tissues (Figure 7b). Moreover, the percentage of global DNA methylation was below 1.2% in all groups of mice. 

#### 3.5.2. Analysis of Histone H4 Modification

**Figure 8:**
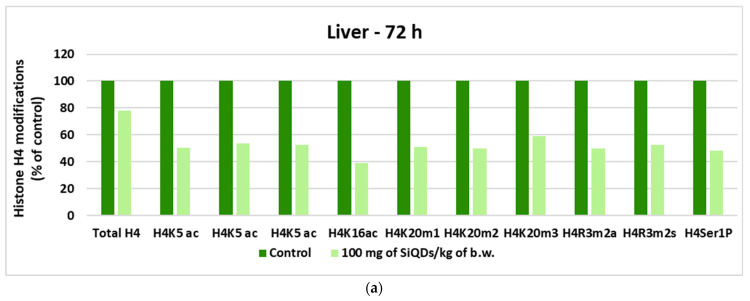

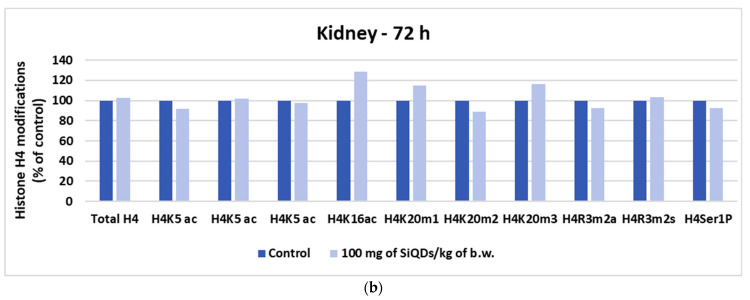
Changes in histone H4 in liver (**a**) and kidney (**b**) samples collected at 72 h after SiQDs administration (100 mg of QDs/kg of b.w.).

Histones are the main protein components of chromatin, playing an important role in gene regulation. H2A, H2B, H3, and H4 represent the major histones, and their post-translational modifications include acetylation of specific lysine residues by acetyl transferases, deacetylation by deacetylases, methylation of arginine and lysine residues by methyl transferases, and demethylation of lysine residues by demethylases [39]. The analysis of histone H4 changes at the liver indicated a global percentage of histone H4 methylation of below 50% for all variants tested (Figure 8a), while at the kidney level, an increase in the degree of methylation is noted only in the case of H4k16ac, H4k20m1, and H4k20m3, with increases of 28%, 15%, and 16%, respectively, above the control level (Figure 8b).

## 4. Discussion

The present study examined the toxicity mechanisms triggered by doses of up to 100 mg of SiQDs/kg of b.w. of SiQDs in mouse livers and kidneys. For this purpose, the assessment of oxidative stress, expression of cell death proteins, genotoxicity, and tissue morphology were carried out at different time points after QDs administration. 

Exposure to QDs may induce oxidative stress through various mechanism that especially involve free radicals. Considering that the superoxide anion is generated in the reaction catalyzed by the membrane NADPH oxidase (NOX) and that the SiQDs are internalized by macropinocytosis [12], it can be assumed that the nanoparticle interaction with the membranes will increase NOX activity. Also, the reaction between water molecules and the SiO_2_ layer of QDs could result in hydrogen peroxide accumulation, leading to CAT depletion or its functional inhibition, with changes in cell function [40]. Exposure to SiQDs can also stimulate the redox signaling pathways in the cell, which amplify the cells’ response to oxidative stress [41].

Our results showed that high doses of SiQDs were able to induce oxidative stress in liver. Although the significant decrease in CAT activity could indicate a high amount of hydrogen peroxide, we should take into consideration that the increase in GPx activity after the first 6 h of exposure could actually suggest that the hydrogen peroxide was maintained at a low level based on the difference in K_M_ for this substrate between these two enzymes. Similar results regarding the hepatic CAT activity after QDs administration have also been reported by Das et al. [42]. The transient increase in SOD activity at 6 h after administration might be due to either indirect activation by SiQDs, stimulating the production of superoxide anion in the cells and activating the antioxidant defense mechanisms in order to counteract the radicals; or due to the alteration of gene expression involved in SOD synthesis. The probable low amount of superoxide anion in liver samples could be correlated with previous data [17,20], indicating the ability of this organ to neutralize the toxic effects produced by SiQDs. 

The hepatic GST activity after the first hour indicated a dose-dependent decrease, pointing out the sensitivity of GST to the effects of oxidative stress produced by the presence of SiQDs. The analysis of GPx activity in the liver indicated a time- and concentration-dependent increase up to the first 6 h, suggesting a stimulation of cell antioxidant capacity following the SiQDs administration. Furthermore, the slight increased values of GPx activity at 6 h correlated with GST activity from this time interval, indicating the activation of the liver’s ability to neutralize the toxic effects of SiQDs. At 24 and 72 h post-administration of a high dose of QDs, only a slight decrease in GPx activity was registered, which is in accordance with other safe QDs such as Mn-doped ZnS QDs-PEG [18] but is in contrast with CdSe QDs’ effect induced in rats (halving of activity) [42] and in mice (three-fold increase) [43]. 

Taking into account that hepatic Gred activity did not show major decreases compared with the control between the 6 and 72 h after QDs administration, it can be explained why the GSH concentration was almost at the same level as control in all exposed mice (Figure 5). Possibly, the significant decrease in hepatic GPx and GST activities after exposure to 100 mg of SiQDs/kg of b.w. could occur in spite of the normal level of GSH, due to the fact that, in mice organism and cells, these QDs could be surrounded by protein corona [44] and as a result could interact with the 3D fold of GPx and GST proteins [45], modifying their activity. 

A transient increase in the MDA level observed at 6 h indicated a slight susceptibility to lipid peroxidation in liver, which is in accordance with the CAT activity decrease. However, the liver successfully neutralized the toxicity after 24 h, and the MDA level returned to the control values. No changes in MDA levels were also measured in the liver of mice that were injected with non-toxic near infrared QD800 linked to peptides containing arginine–glycine–aspartic acid [17] and Mn-doped ZnS QDs and QDs-PEG [18]; however, significant higher lipid peroxidation in hepatic cells was previously obtained for the toxic CdTe QDs [19,20] and CdSe QDs [42,43]. 

Furthermore, the results on kidney tissue showed that exposure to SiQDs in high doses generated toxic effects that were much more pronounced than in liver. The decrease in CAT activity in a dose-dependent manner could indicate a reduction in its function due to hydrogen peroxide generation and accumulation. In contrast, more toxic QDs, such as CdTe and CdSe QDs, induced higher CAT activities in mice [19] and rats [42], respectively, compared with the control. After a dose-dependent decrease in SOD activity after the first hour, an increase in this enzyme’s activity compared with the control was measured after 6 h, suggesting that SOD was vulnerable at the beginning of exposure due to superoxide anion generation; however, antioxidant defense mechanisms were further activated to neutralize the free radicals. Nevertheless, this mechanism was not enough to counteract the oxidative stress as we could observe a decrease in SOD activity after 24 and 72 h. A previous report of Das et al. [42] showed the same reduction in renal SOD activity after CdSe QD administration at a high dose to rats. This pattern of activity dynamics was also evidenced in the case of Gred and GPx, showing that the balance between GSH utilization and regeneration was perturbed, as it was confirmed by the dose- and time-dependent decrease in GSH levels. These results highlighted the kidney’s attempts to counteract the toxic effects of SiQDs, but the neutralization potential decreased after 24 h. However, Wang et al. [19] showed a higher depletion in the renal GSH level after CdTe QD administration to mice, confirming that SiQDs are a safer choice. Also, the same aspect as in our study regarding the low GSH levels in kidney compared with liver concentrations was noticed by Wang et al. [19]. 

The renal toxicity of 100 mg of QDs/kg of b.w. is evidenced by the changes in the activity of GST, which showed an increase after 24 h followed by a decrease at 72 h, most probably being activated in response to the exogenous electrophilic compound, and it was followed by a decline as a consequence of GSH diminution. However, the lipid peroxidation at renal level did not exceed more than 117% of control after 72 h and confirmed that these SiQDs did not induce a high toxicity as was described for CdSe QDs, which doubled the MDA level after the administration of 40 mg of QDs/kg to Wistar rats [42]. Most probably, the higher toxicity in the kidney compared with the liver for the highest dose of QDs tested is driven by the process of urine concentration within this organ and, therefore, of toxicant accumulation. The most important consequence induced by free radicals formed after SiQDs administration in high concentrations is the oxidative damage to cell membranes, ultimately causing the dysfunction of the affected organ. 

Previously, it was reported that there was a rapid clearance (1 h) of dextran-coated SiQDs from the mouse bloodstream, the QDs being excreted by renal filtration and urinary bladder, and some of them accumulated in the liver after 48 h [46]. Also, PEGylated Ag_2_Se QDs especially accumulated in the spleen and liver and were cleared within 1 day [47]. Furthermore, a blood circulation half-life of about 7 h was obtained for PEG-CSQDs [48]. As a general trend, nanoparticles with a size smaller than 7 nm should be fast and efficiently eliminated via renal filtration with excretion into the urine, while this renal clearance will decrease for the bigger particles, which will be transported to the liver, spleen, and lymphatic system and further excreted into the biliary system and intestine [46].

The histopathological analysis indicated the most significant morphology changes in the kidney and liver tissues and immunopositivity for TNF-α 6 h after SiQDs administration, the degree of modification being dependent on the dose tested. Regarding the extent of inflammation over time, Gerasimovich et al. [49] showed that the serum level of the majority of pro-inflammatory cytokines, including TNF, was similar to that of control 7 days after injection with CdSe/ZnS QDs, suggesting that the immune reaction to QDs is reversible. Hepatic impairments, such as disordered hepatic cords and enlarged central veins, were also detected after CdSe QDs administration [43], and apoptotic and necrotic cells with an increased level of TNF-α were previously shown after only 6 mg/kg of b.w. CdTe QDs injection in mice [50]. In addition, significant kidney injury, represented by hemocytes and necrosis, was reported in CdTe/CdS QDs 655-treated mice [51]. In contrast, Liu et al. [52] showed no toxicity in the body weight or blood chemistry at doses of up to 200 mg of SiQDs/kg of b.w., and Zhang et al. [53] did not evidence toxicity after intravenous administration of 75 mg graphene QDs/kg of b.w. to mice. Therefore, it was expected that SiQDs would have a lower toxicity grace to their physicochemical properties, such as no heavy metals in the structure.

Moreover, SiQDs could have a potential impact on lung tissue given its critical role as a primary target for thrombosis and vasculitis following intravenous administration. Although it was previously reported that CdSe/ZnS QDs caused marked vascular thrombosis in the pulmonary circulation [54], it was also shown that non-cadmium QDs, such as InP/ZnS QDs, induced histopathological abnormalities in mice lungs [55]. Therefore, future complex work is needed to explore the pathological features, mechanisms of action, and potential clinical significance of SiQDs-induced changes in the lungs.

The increase in Nrf-2 protein expression after the administration of 100 mg of SiQDs/kg of b.w. in mice correlated with the activity of hepatic GPx and SOD; most probably, the upregulation of Nrf-2 expression activated the antioxidant defense system as a reaction to the presence of SiQDs. The lack of change in the relative expression of Beclin-1 protein indicated that autophagic processes were not activated. The increase in LC-3 levels after the first hour of exposure could be explained by the fact that this protein can be overexpressed when intracellular protein aggregates are formed or when QD aggregates are sequestered in vacuoles, with similar results being reported previously [32]. Also, the increase in the level of LC-3 can suggest the reduction of autophagic processes [56]. The analysis of p53 protein expression indicated an interesting contrast after 6 h between the liver and kidney tissues. The decrease in p53 in hepatic cells suggested the cellular potential of regeneration, and the upregulation of p53 in renal cells confirmed the apoptosis, as was also confirmed by morphology changes revealed in histopathology images. 

Generally, the genotoxicity induced by QDs is influenced by the particle dose, exposure time, and their physicochemical properties, which regulate the interaction with biomolecules [57]. Direct genotoxicity involves the interaction of particles or ions released from them with the genetic material or damage to the genetic material by ROS produced by intracellular QDs. On the other hand, when the QDs are not internalized, the indirectly induced genotoxicity occurs, especially during the inflammatory response induced by QDs when ROS produced by activated neutrophils or macrophages carry out oxidative attacks on DNA molecules [58,59]. Our analysis regarding the genotoxicity of SiQDs (100 mg/kg of b.w.) after exposure for 72 h indicated no significant genotoxicity in both types of analyzed tissues. Considering that only few studies have investigated the effect of nanoparticles on DNA methylation, it is difficult to compare the findings with the available literature. There are previous reports on DNA hypomethylation in human keratinocytes HaCaT after incubation with SiO_2_ nanoparticles [60] in the lungs and blood of mice exposed by airways to multiwall carbon nanotubes [61], suggesting an association with oxidative stress and activation of epigenetic mechanisms of cell repair [62]. On the other hand, global DNA methylation increased in the lungs of mice instilled with copper oxide nanoparticles [63] and in the livers of zebrafish after exposure to aminated graphene QDs [64]. A similar decrease in global H4K5ac in the liver of mice that received SiQDs (Figure 8a) was observed after treatment of human keratinocytes with ZnO nanoparticles, suggesting that the nucleus was stressed, and the histone lysine residue modifications gave signals for the recruitment of DNA repair proteins and chromatin structure remodeling [65].

## 5. Conclusions

This study provided a full evaluation of the in vivo impact of SiQDs synthesized by laser ablation on oxidative stress, cell death, and epigenetic changes. We found out that the main effect of SiQDs in liver and kidneys was the generation of oxidative stress, which occurred when doses were higher than 10 mg of QD/kg of b.w. Also, the administration of a high dose of SiQDs induced necrosis and apoptosis at the tissue level, with significant changes in tissue morphology and immunopositivity for TNF-α, which was particularly more intense for kidneys. However, there was no significant genotoxicity evidenced in the analyzed tissues. In conclusion, our data provide useful information for further clinical studies as we showed that doses lower than 10 mg of SiQDs/kg of b.w. did not induce hepatic and renal toxicity; however, further work is needed to evaluate the SiQDs effects on other organs, such as lungs. 

## Data Availability

Data are available upon request from the corresponding authors.
