# Correction: Cristian et al. In Vivo Assessment of Hepatic and Kidney Toxicity Induced by Silicon Quantum Dots in Mice. *Nanomaterials* 2024, *14*, 457

**DOI:** 10.3390/nano15241857

**Published:** 2025-12-11

**Authors:** Roxana-Elena Cristian, Cornel Balta, Hildegard Herman, Bogdan Trica, Beatrice G. Sbarcea, Anca Hermenean, Anca Dinischiotu, Miruna S. Stan

**Affiliations:** 1 Departament of Biochemistry and Molecular Biology, Faculty of Biology, University of Bucharest, 91-95 Splaiul Independentei, 050095 Bucharest, Romania; roxana.cristian@drd.unibuc.ro (R.-E.C.); anca.hermenean@gmail.com (A.H.); miruna.stan@bio.unibuc.ro (M.S.S.); 2 DANUBIUS Department, National Institute of Research and Development for Biological Sciences, Splaiul Independentei 296, 060031 Bucharest, Romania; 3 “Aurel Ardelean” Institute of Life Sciences, Vasile Goldis Western University of Arad, 86 Rebreanu, 310414 Arad, Romania; balta.cornel@uvvg.ro (C.B.); herman.hildegard@uvvg.ro (H.H.); 4 National Institute for Research & Development in Chemistry and Petrochemistry (INCDCP-ICECHIM), 202 Spl. Independentei, 060021 Bucharest, Romania; trica.bogdan@gmail.com; 5 Materials Characterization Department, National Institute for Research & Development in Electrical Engineering (ICPE-CA), 313 Splaiul Unirii, 030138 Bucharest, Romania; gabriela.sbarcea@icpe-ca.ro; 6 Research Institute of the University of Bucharest (ICUB), University of Bucharest, 91-95 Spl. Independentei, 050095 Bucharest, Romania

In the original publication [1], there was a mistake in Figure 2. The error in the published article resulted from the inadvertent selection of incorrect images from our storage folders during the figure assembly process, namely histopathological images of liver tissue samples at 1 and 72 h treated with 10 mg of SiQDs/kg and at 24 h treated with 1 mg SiQDs/kg, as well as subfigures of histopathological images of kidney tissue samples at 1 h with 10 mg of SiQDs/kg and 6 h with 1 mg SiQDs/kg.

The corrected Figure 2 appears below:

**Figure 2:**
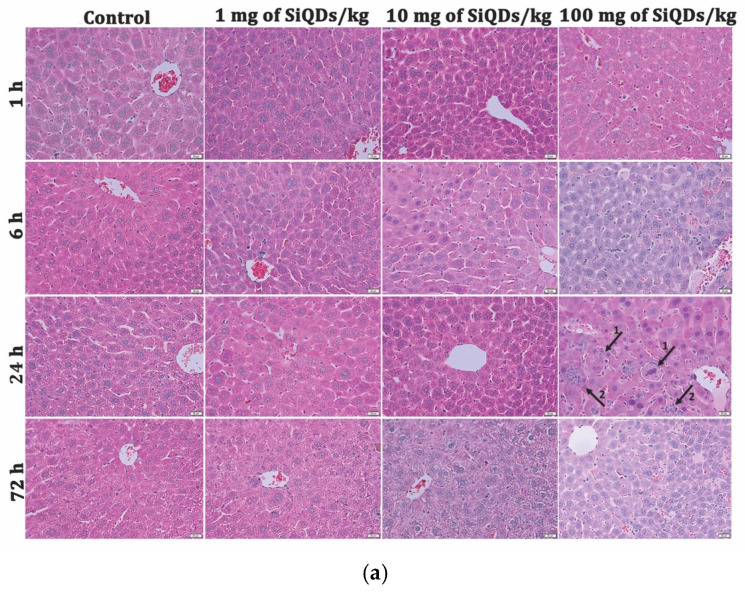

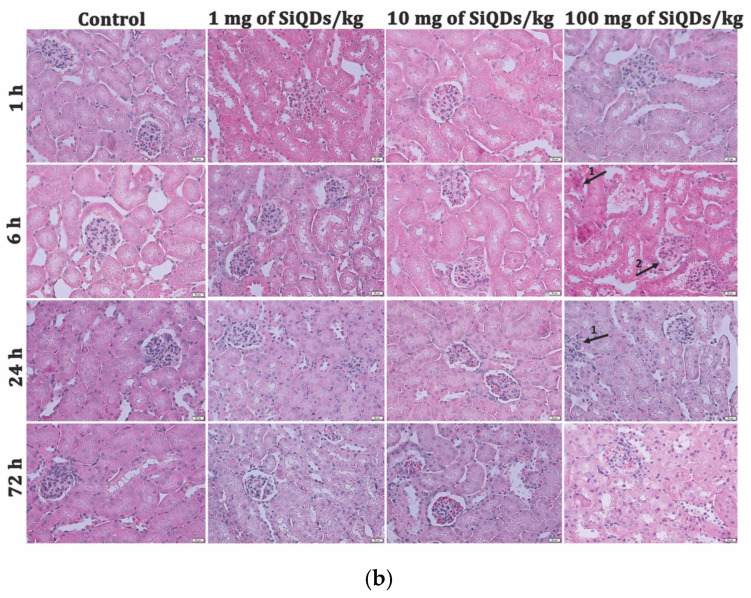
Liver (**a**) and kidney (**b**) histopathology at 1 h, 6 h, 24 h, and 72 h after SiQDs administration (1, 10, and 100 mg/kg of b.w.) in mice. Tissue sections (5 µm) were stained with hematoxylin and eosin and examined by light microscopy. Legend of arrows: (**a**) 1—hepatocyte swelling, 2—inflammatory infiltrates; (**b**) 1—vascular congestion; 2—glomerular atrophy. Magnification of 10× for liver images and 20× for kidney images.

The authors state that the scientific conclusions are unaffected. This correction was approved by the Academic Editor. The original publication has also been updated.
